# An improved filtering algorithm for big read datasets and its application to single-cell assembly

**DOI:** 10.1186/s12859-017-1724-7

**Published:** 2017-07-03

**Authors:** Axel Wedemeyer, Lasse Kliemann, Anand Srivastav, Christian Schielke, Thorsten B. Reusch, Philip Rosenstiel

**Affiliations:** 1 Department of Computer Science, Kiel University, Christian-Albrechts-Platz 4, Kiel, 24118 Germany; 2 Marine Ecology, GEOMAR Helmholtz Centre for Ocean Research Kiel, Düsternbrooker Weg 20, Kiel, 24105 Germany; 3 Institute of Clinical Molecular Biology, Kiel University, Schittenhelmstr. 12, Kiel, 24105 Germany

**Keywords:** Read filtering, Read normalization, Bignorm, Diginorm, Singe cell sequencing, Coverage

## Abstract

**Background:**

For single-cell or metagenomic sequencing projects, it is necessary to sequence with a very high mean coverage in order to make sure that all parts of the sample DNA get covered by the reads produced. This leads to huge datasets with lots of redundant data. A filtering of this data prior to assembly is advisable. Brown et al. (2012) presented the algorithm Diginorm for this purpose, which filters reads based on the abundance of their *k*-mers.

**Methods:**

We present Bignorm, a faster and quality-conscious read filtering algorithm. An important new algorithmic feature is the use of phred quality scores together with a detailed analysis of the k-mer counts to decide which reads to keep.

**Results:**

We qualify and recommend parameters for our new read filtering algorithm. Guided by these parameters, we remove in terms of median 97.15% of the reads while keeping the mean phred score of the filtered dataset high. Using the SDAdes assembler, we produce assemblies of high quality from these filtered datasets in a fraction of the time needed for an assembly from the datasets filtered with Diginorm.

**Conclusions:**

We conclude that read filtering is a practical and efficient method for reducing read data and for speeding up the assembly process. This applies not only for single cell assembly, as shown in this paper, but also to other projects with high mean coverage datasets like metagenomic sequencing projects.

Our Bignorm algorithm allows assemblies of competitive quality in comparison to Diginorm, while being much faster. Bignorm is available for download at https://git.informatik.uni-kiel.de/axw/Bignorm.

**Electronic supplementary material:**

The online version of this article (doi:10.1186/s12859-017-1724-7) contains supplementary material, which is available to authorized users.

## Background

Next generation sequencing systems (such as the Illumina platform) tend to produce an enormous amount of data — especially when used for single-cell or metagenomic protocols — of which only a small fraction is essential for the assembly of the genome. It is thus advisable to filter that data prior to assembly.

A coverage of about 20 for each position of the genome has been empirically determined as optimal for a successful assembly of the genome [1]. On the other hand, in many setups, the coverage for a large number of loci is much higher than 20, often rising up to tens or hundreds of thousands, especially for single-cell or metagenomic protocols (see Table 1, “max” column for the maximal coverage of the datasets that we use in our experiments). In order to speed up the assembly process — or in extreme cases to make it possible in the first place, given certain restrictions on available RAM and/or time — a sub-dataset of the sequencing dataset is to be determined such that an assembly based on this sub-dataset works as good as possible. For a formal description of the problem, see Additional file 1: Section S1.

**Table 1:** Coverage statistics for Bignorm with *Q*_0_=20, Diginorm, and the raw datasets

Dataset	Algorithm	$\mathcal {P}10$	Mean	$\mathcal {P}90$	Max
Aceto	Bignorm	6	132	216	6801
	Diginorm	7	171	295	12,020
	Raw	15	9562	17,227	551,000
Alphaproteo	Bignorm	10	43	92	884
	Diginorm	7	173	481	6681
	Raw	25	5302	14,070	303,200
Arco	Bignorm	1	98	54	2103
	Diginorm	1	362	200	6114
	Raw	3	10,850	4091	220,600
Arma	Bignorm	8	23	32	358
	Diginorm	8	79	141	5000
	Raw	17	629	1118	31,260
ASZN2	Bignorm	40	70	83	2012
	Diginorm	23	143	354	3437
	Raw	50	1738	4784	43,840
Bacteroides	Bignorm	3	74	90	6768
	Diginorm	3	123	205	7933
	Raw	7	6051	8127	570,900
Caldi	Bignorm	25	63	110	786
	Diginorm	15	67	135	3584
	Raw	27	1556	3643	33,530
Caulo	Bignorm	7	228	216	10,400
	Diginorm	8	362	491	35,520
	Raw	8	10,220	9737	464,300
Chloroflexi	Bignorm	8	72	101	2822
	Diginorm	9	412	878	20,850
	Raw	9	5612	7741	316,900
Crenarch	Bignorm	8	104	159	3770
	Diginorm	10	560	1285	29,720
	Raw	10	8086	14,987	316,700
Cyanobact	Bignorm	9	144	153	5234
	Diginorm	10	756	1450	26,980
	Raw	10	9478	11,076	356,600
E.coli	Bignorm	37	45	56	234
	Diginorm	50	382	922	7864
	Raw	112	2522	6378	56,520
SAR324	Bignorm	24	49	71	1410
	Diginorm	18	53	107	2473
	Raw	26	1086	2761	106,000

### Previous work

We briefly survey two prior approaches for read pre-processing, namely *trimming* and *error correction*. Read trimming programs (see [2] for a recent review) try to cut away the low quality parts of a read (or drop reads whose overall quality is low). These algorithms can be classified into two groups: *running sum* (Cutadapt, ERNE, SolexaQA with -bwa option [3–5]) and *window based* (ConDeTri, FASTX, PRINSEQ, Sickle, SolexaQA, and Trimmomatic [5–10]). The running sum algorithms take a quality threshold *Q* as input, which is subtracted from the phred score of each base of the read. The algorithms vary with respect to the functions applied to these differences to determine the quality of a read, the direction in which the read is processed, the function’s quality threshold upon which the cutoff point is determined, and the minimum length of a read after the cutoff to be accepted.

The window based algorithms, on the other hand, first cut away the reads’s 3’ or 5’ ends (depending on the algorithm) whose quality is below a specified minimum quality parameter and then determine a contiguous sequence of high quality using techniques similar to those used in the running sum algorithms.

All of these trimming algorithms generally work on a per-read basis, reading the input once and processing only a single read at a time. The drawback of this approach is that low quality sequences within a read are being dropped even when these sequences are not covered by any other reads whose quality is high. On the other hand, sequences whose quality and abundance are high are added over and over although their coverage is already high enough, which yields higher memory usage than necessary.

Most of the error correction programs (see [11] for a recent review) read the input twice: a first pass gathers statistics about the data (often *k*-mer counts) which in a second pass are used to identify and correct errors. Some programs trim reads which cannot be corrected. Again, coverage is not a concern: reads which seem to be correct or which can be corrected are always accepted. According to [11], currently the best known and most used error correction program is Quake [12]. Its algorithm is based on two assumptions:

- “For sufficiently large *k*, almost all single-base errors alter *k*-mers overlapping the error to versions that do not exist in the genome. Therefore, *k*-mers with low coverage, particularly those occurring just once or twice, usually represent sequencing errors.”
- Errors follow a Gamma distribution, whereas true *k*-mers are distributed as per a combination of the Normal and the Zeta distribution.

In the first pass of the program, a score based on the phred quality scores of the individual nucleotides is computed for each *k*-mer. After this, Quake computes a *coverage cutoff* value, that is, the local minimum of the *k*-mer spectrum between the Gamma and the Normal maxima. All *k*-mers having a score higher than the coverage cutoff are considered to be correct (*trusted* or *solid* in error correction terminology), the others are assumed to be erroneous. In a second pass, Quake reads the input again and tries to replace erroneous *k*-mers by trusted ones using a maximum likelihood approach. Reads which cannot be corrected are optionally trimmed or dumped.

But the main goal of error correctors is not the reduction of the data volume (in particular, they do not pay attention to excessive coverage), hence they cannot replace the following approaches.

Brown et al. invented an algorithm named *Diginorm* [1, 13] for read filtering that rejects or accepts reads based on the abundance of their *k*-mers. The name *Diginorm* is a short form for *digital normalization*: the goal is to normalize the coverage over all loci, using a computer algorithm after sequencing. The idea is to remove those reads from the input which mainly consist of *k*-mers that have already been observed many times in other reads. Diginorm processes reads one by one, splits them into *k*-mers, and counts these *k*-mers.

In order to save RAM, Diginorm does not keep track of those numbers exactly, but instead keeps appropriate estimates using the count-min sketch (CMS [14], see Additional file 1: Section S1.2 for a formal description). A read is accepted if the median of its *k*-mer counts is below a fixed threshold, usually 20. It was demonstrated that successful assemblies are still possible after Diginorm removed the majority of the data.

### Our algorithm — Bignorm

Diginorm is a pioneering work. However, the following points, which are important from the biological or computational point of view, are not covered in Diginorm. We consider them as the algorithmic innovation in our work:

(i) We incorporate the important phred quality score into the decision whether to accept or to reject a read, using a quality threshold. This allows a tuning of the filtering process towards high-quality assemblies by using different thresholds.
(ii) When deciding whether to accept or to reject a read, we do a detailed analysis of the numbers in the count vectors. Diginorm merely considers their medians.
(iii) We offer a better handling of the N case, that is, when the sequencing machine could not decide for a particular nucleotide. Diginorm simply converts all N to A, which can lead to false *k*-mer counts.
(iv) We provide a substantially faster implementation. For example, we include fast hashing functions (see [15, 16]) for counting *k*-mers through the count-min sketch data structure (CMS), and we use the C programming language and OpenMP.

A technical description of our algorithm, called *Bignorm*, is given in Additional file 1: Section S1.3, which might be important for computer scientists and mathematicians working in this area.

## Methods

### Experimental setup

For the experimental evaluation, we collected the following datasets. We use two single cell datasets of the UC San Diego, one of the group of Ute Hentschel (now GEOMAR Kiel) and 10 datasets from the JGI Genome Portal. The datasets from JGI were selected as follows. On the JGI Genome Portal [17], we used “single cell” as search term. We narrowed the results down to datasets with all of the following characteristics:

- status “complete”;
- containing read data *and* an assembly in the download section;
- aligning the reads to the assembly using Bowtie 2 [18] yields an “overall alignment rate” of more than 70*%*.

From those datasets, we arbitrarily selected one per species, until we had a collection of 10 datasets. We refer to each combination of species and selected dataset as a *case* in the following. In total, we have 13 cases; the details are given in Table 2.

**Table 2:** Selected species and datasets (Cases)

Short name	Species/Description	Source	URL
ASZN2	Candidatus Poribacteria sp. WGA-4E_FD	Hentschel Group [27]	[28]
Aceto	Acetothermia bacterium JGI MDM2 LHC4sed-1-H19	JGI Genome Portal	[29]
Alphaproteo	Alphaproteobacteria bacterium SCGC AC-312_D23v2	JGI Genome Portal	[30]
Arco	Arcobacter sp. SCGC AAA036-D18	JGI Genome Portal	[31]
Arma	Armatimonadetes bacterium JGI 0000077-K19	JGI Genome Portal	[32]
Bacteroides	Bacteroidetes bacVI JGI MCM14ME016	JGI Genome Portal	[33]
Caldi	Calescamantes bacterium JGI MDM2 SSWTFF-3-M19	JGI Genome Portal	[34]
Caulo	Caulobacter bacterium JGI SC39-H11	JGI Genome Portal	[35]
Chloroflexi	Chloroflexi bacterium SCGC AAA257-O03	JGI Genome Portal	[36]
Crenarch	Crenarchaeota archaeon SCGC AAA261-F05	JGI Genome Portal	[37]
Cyanobact	Cyanobacteria bacterium SCGC JGI 014-E08	JGI Genome Portal	[38]
E.coli	E.coli K-12, strain MG1655, single cell MDA, Cell one	UC San Diego	[39]
SAR324	SAR324 (Deltaproteobacteria)	UC San Diego	[39]

For each case, we analyze the results obtained with Diginorm and with Bignorm using quality parameters *Q*_0_∈{5,8,10,12,15,18,20,…,45}. Analysis is done on the one hand in terms of data reduction, quality, and coverage. On the other hand, we study actual assemblies that are computed with SPAdes [19] based on the raw and filtered datasets. For comparison, we also did assemblies using IDBA_UD [20] and Velvet-SC [21] (for *Q*_0_=20 only). All the details are given in the next section.

The dimensions of the count-min sketch are fixed to *m*=1,024 and *t*=10, thus 10 GB of RAM were used.

## Results

For our analysis, we mainly considered percentiles and quartiles of measured parameters. The *i*th quartile is denoted by $\mathcal {Q}i$, where we use $\mathcal {Q}0$ for the minimum, $\mathcal {Q}2$ for the median, and $\mathcal {Q}4$ for the maximum. The *i*th percentile is denoted by $\mathcal {P}i$; we often use the 10th percentile $\mathcal {P}10$.

### Number of accepted reads

**Fig. 1:**
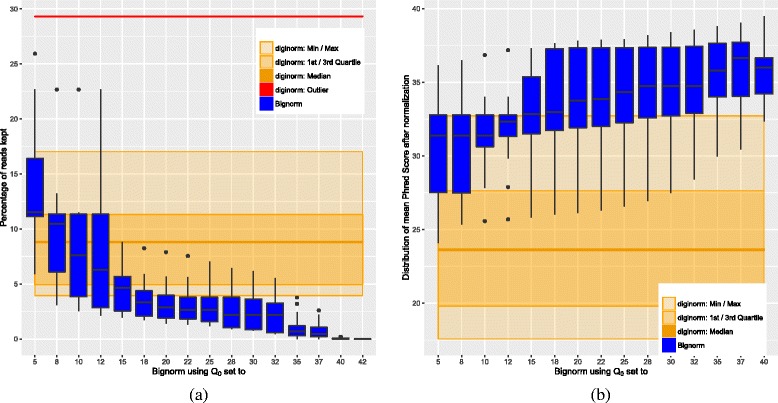
Box plots showing reduction and quality statistics. **a** Percentage of accepted reads (i.e. reads kept) over all datasets. **b** Mean quality values of the accepted reads over all datasets

Statistics for the number of accepted reads are given as a box plot in Fig. 1 a. This plot is constructed as follows. Each of the blue boxes corresponds to Bignorm with a particular *Q*_0_, while Diginorm is represented as the wide orange box in the background (recall that Diginorm does not consider quality values). Note that the “whiskers” of Diginorm’s box are shown as light-orange areas. For each box, for each case the raw dataset is filtered using the algorithm and algorithmic parameters corresponding to that box, and the percentage of the accepted reads is taken into consideration. For example, if the top of a box (which corresponds to the 3rd quartile, also denoted $\mathcal {Q}3$) gives the value *x* *%*, then we know that for 75*%* of the cases, *x* *%* or less of the reads were accepted using the algorithm and algorithmic parameters corresponding to this box.

There are two prominent outliers: one for Diginorm with value ≈29*%* (shown as the red line at the top) and one for Bignorm for *Q*_0_=5 with value ≈26*%*. In both cases, the Arma dataset is responsible, which is the dataset with the worst mean phred score and the strongest decline of the phred score over the read length (see Additional file 1: Section S4 for more information and per base sequence quality plots). This suggest that the high rate of read kept is caused by a high error rate of the dataset. For 15≤*Q*_0_, even Bignorm’s outliers fall below Diginorm’s median, and for 18≤*Q*_0_ Bignorm keeps less than 5*%* of the reads for at least 75*%* of the datasets. In the range 20≤*Q*_0_≤25, Bignorm delivers similar results for the different values of *Q*_0_, and the gain in reduction for larger *Q*_0_ is small up to *Q*_0_=32. For even larger *Q*_0_, there is another jump in reduction, but we will see that coverage and the quality of the assembly suffer too much in that range. We conjecture that in the range 18≤*Q*_0_≤32, we remove most of the actual errors, whereas for larger *Q*_0_, we also remove useful information.

### Quality values

Statistics for phred quality scores in the filtered datasets are given in Fig. 1. The data was obtained using fastx_quality_stats from the FASTX Toolkit [7] on the filtered fastq files and calculating the mean phred quality scores over all read positions for each dataset. Looking at the statistics for these overall means, for 15≤*Q*_0_, Bignorm’s median is better than Diginorm’s maximum. For 20≤*Q*_0_, this effect becomes even stronger. For all values for *Q*_0_, Bignorm’s minimum is clearly above Diginorm’s median. Note that an increase of 10 units means reducing error probability by factor 10.

In Table 3, we give quartiles of mean quality values for the raw datasets and Bignorm’s datasets produced with *Q*_0_=20. Bignorm improves slightly on the raw dataset in all five quartiles.

**Table 3:** Comparing quality values for the raw dataset and Bignorm with *Q*_0_=20

Quartile	Bignorm	Raw
$\mathcal {Q}4$ (max)	37.82	37.37
$\mathcal {Q}3$	37.33	36.52
$\mathcal {Q}2$ (median)	33.77	32.52
$\mathcal {Q}1$	31.91	30.50
$\mathcal {Q}0$ (min)	26.14	24.34

Of course, all this could be explained by Bignorm simply cutting away any low-quality reads. However, the data in the next section suggests that Bignorm may in fact be more careful than this.

### Coverage

**Fig. 2:**
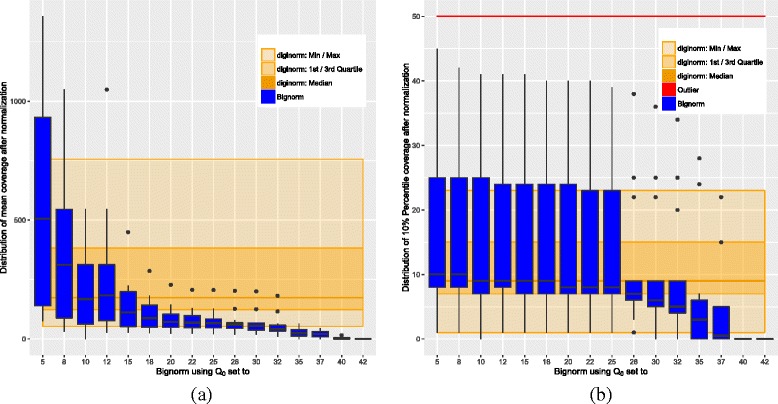
Box plots showing coverage statistics. **a** Mean coverage over all datasets. **b** 10th percentile of the coverage over all datasets

In Fig. 2, we see statistics for the coverage. The data was obtained by remapping the filtered reads onto the assembly from the JGI using Bowtie 2 and then using coverageBed from the bedtools [22] and R [23] for the statistics. In Fig. 2 a, the mean is considered. For 15≤*Q*_0_, Bignorm reduces the coverage heavily. For 20≤*Q*_0_, Bignorm’s $\mathcal {Q}3$ is below Diginorm’s $\mathcal {Q}1$. This may raise the concern that Bignorm could create areas with insufficient coverage. However, in Fig. 2 b, we look at the 10th percentile ($\mathcal {P}10$) of the coverage instead of the mean. We consider this statistics as an indicator for the impact of the filtering on areas with low coverage. For *Q*_0_≤25, Bignorm’s $\mathcal {Q}3$ is at or above Diginorm’s maximum, and Bignorm’s minimum coincides with Diginorm’s (except for *Q*_0_=10, where we are slightly below). In terms of the median, both algorithms are very similar for *Q*_0_≤25. We consider all this as a strong indication that we cut away in the right places.

For 28≤*Q*_0_, there is a clear drop in coverage, so we do not recommend such *Q*_0_ values.

In Table 1, we give coverage statistics for each dataset. The reduction compared to the raw dataset in terms of mean, $\mathcal {P}90$, and maximum is substantial. But also the improvement of Bignorm over Diginorm in mean, $\mathcal {P}90$, and maximum is considerable for most datasets.

### Assessment through assemblies

**Fig. 3:**
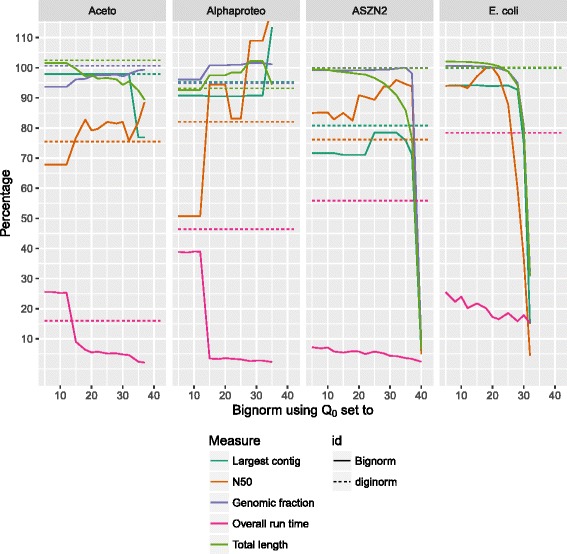
Assembly statistics for four selected datasets; measurements of assemblies performed on the datasets with prior filtering using Diginorm and Bignorm, relative to the results of assemblies performed on the unfiltered datasets

The quality and significance of read filtering is subject to complete assemblies, which is the final “road test” for these algorithms. For each case, we do an assembly with SPAdes using the raw dataset and those filtered with Diginorm and Bignorm for a selection of *Q*_0_ values. The assemblies are then analyzed using quast [24] and the assembly from the JGI as reference. Statistics for four cases are shown in Fig. 3. We give the quality measures N50, genomic fraction, and largest contig, and in addition the overall running time (pre-processing plus assembler Wall time). Each measure is given in percentage relative to the raw dataset.

Generally, our biggest improvements are for N50 and running time. For 15≤*Q*_0_, Bignorm is always faster than Diginorm, for three of the four cases by a large margin. In terms of N50, for 15≤*Q*_0_, we observe improvements for three cases. For E.coli, Diginorm’s N50 is 100*%*, that we also attain for *Q*_0_=20. In terms of genomic fraction and largest contig, we cannot always attain the same quality as Diginorm; the biggest deviation at *Q*_0_=20 is 10 percentage points for the ASZN2 case. The N50 is generally accepted as one of the most important measures, as long as the assembly represents the genome well (as measured by the genomic fraction here) [25].

In Tables 4 and 5, we give statistics for *Q*_0_=20 and each dataset. In terms of genomic fraction, Bignorm is generally not as good as Diginorm. However, excluding the Aceto and Arco cases, Bignorm’s genomic fraction is still always at least 95*%*. For Aceto and Arco, Bignorm misses 3.21*%* and 3.48*%*, respectively, of the genome in comparison to Diginorm. In 8 cases, Bignorm’s N50 is better or at least as good as Diginorm’s. The 4 cases where we achieved a smaller N50 are Arco, Caldi, Caulo, Crenarch, and Cyanobact.

**Table 4:** Filter and assembly statistics for Bignorm with *Q*_0_=20, Diginorm, and the raw datasets (Part I)

Dataset	Algorithm	Reads kept	Mean phred	Contigs	Filter time	SPAdes time
		in %	score	≥10 000	in sec	in sec
Aceto	Bignorm	3.16	37.33	1	906	1708
	Diginorm	3.95	27.28	1	3290	4363
	Raw		36.52	3		47,813
Alphaproteo	Bignorm	3.13	34.65	18	623	420
	Diginorm	7.81	28.73	17	1629	11,844
	Raw		33.64	17		29,057
Arco	Bignorm	2.20	33.77	4	429	207
	Diginorm	8.76	21.39	6	1410	1385
	Raw		32.27	6		15,776
Arma	Bignorm	7.90	28.21	44	240	135
	Diginorm	29.30	21.19	50	588	1743
	Raw		26.96	44		5371
ASZN2	Bignorm	5.66	37.66	118	1224	1537
	Diginorm	12.62	32.73	130	5125	21,626
	Raw		36.85	112		47,859
Bacteroides	Bignorm	2.85	37.47	6	653	3217
	Diginorm	4.94	27.64	5	2124	3668
	Raw		37.25	9		32,409
Caldi	Bignorm	3.97	37.82	41	842	455
	Diginorm	5.61	30.67	36	1838	793
	Raw		37.37	38		7563
Caulo	Bignorm	2.40	36.95	10	679	712
	Diginorm	4.70	25.16	9	2584	765
	Raw		36.01	13		18,497
Chloroflexi	Bignorm	1.40	31.91	32	694	134
	Diginorm	9.70	18.91	33	2304	1852
	Raw		30.50	34		15,108
Crenarch	Bignorm	1.46	33.18	19	1107	790
	Diginorm	9.72	19.80	18	2931	3754
	Raw		31.49	26		20,590
Cyanobact	Bignorm	1.65	30.45	12	679	450
	Diginorm	11.30	17.58	13	1487	1343
	Raw		28.49	13		9417
E. coli	Bignorm	1.91	26.14	67	2279	598
	Diginorm	17.03	19.34	63	9105	3995
	Raw		24.34	64		16,706
SAR324	Bignorm	4.34	33.05	55	1222	708
	Diginorm	4.69	23.58	52	3706	3085
	Raw		32.52	51		26,237

**Table 5:** Filter and assembly statistics for Bignorm with *Q*_0_=20, Diginorm, and the raw datasets (Part II)

Dataset	Algorithm	N50			Longest contig length			Genomic fraction			Misassembled contig length		
		abs	% of raw	% of Diginorm	abs	% of raw	% of Diginorm	abs	% of raw	% of Diginorm	abs	% of raw	% of Diginorm
Aceto	Bignorm	2324	79	105	11,525	98	100	91	97	97	52,487	148	178
	Diginorm	2216	76		11,525	98		94	100		29,539	84	
	Raw	2935			11,772			94			35,351		
Alphaproteo	Bignorm	11,750	94	115	43,977	91	95	98	101	105	52,001	120	89
	Diginorm	10,213	82		46,295	95		93	95		58,184	134	
	Raw	12,446			48,586			98			43,388		
Arco	Bignorm	3320	81	97	12,808	57	57	85	100	97	76,797	99	91
	Diginorm	3434	84		22,463	100		88	103		84,613	109	
	Raw	4092			22,439			85			77,888		
Arma	Bignorm	18,432	102	107	108,140	100	100	98	100	100	774,291	91	103
	Diginorm	17,288	96		108,498	100		98	100		748,560	88	
	Raw	18,039			108,498			98			849,085		
ASZN2	Bignorm	19,788	91	88	72,685	71	88	97	99	99	2,753,167	94	105
	Diginorm	16,591	76		82687	81		97	100		2,617,095	89	
	Raw	21,784			102,287			97			2,941,524		
Bacteroides	Bignorm	3356	68	100	25,300	100	100	95	98	99	70,206	105	112
	Diginorm	3356	68		25,300	100		96	99		62,882	94	
	Raw	4930			25,299			98			66,626		
Caldi	Bignorm	50,973	82	83	143,346	89	91	100	100	100	573,836	94	68
	Diginorm	61,108	98		157,479	98		100	100		839,126	138	
	Raw	62,429			160,851			100			609,604		
Caulo	Bignorm	4515	69	95	20,255	100	107	96	98	98	60,362	86	113
	Diginorm	4729	72		18,907	93		98	101		53,456	76	
	Raw	6562			20,255			97			70,161		
Chloroflexi	Bignorm	13,418	102	109	79,605	102	102	99	100	100	666,519	95	93
	Diginorm	12,305	93		78,276	100		100	100		716,473	102	
	Raw	13,218			78,276			99			703,171		
Crenarch	Bignorm	6538	77	91	31,401	81	66	97	99	99	484,354	89	95
	Diginorm	7148	84		47,803	124		98	100		510,256	94	
	Raw	8501			38,582			98			544,763		
Cyanobact	Bignorm	5833	95	99	33,462	98	100	99	101	100	236,391	113	110
	Diginorm	5907	96		33,516	98		99	101		214,574	103	
	Raw	6130			34,300			98			209,269		
E. coli	Bignorm	112,393	100	100	268,306	94	94	96	100	100	28,966	65	65
	Diginorm	112,393	100		285,311	100		96	100		44,465	100	
	Raw	112,393			285,528			96			44,366		
SAR324	Bignorm	135,669	100	114	302,443	100	100	99	100	100	4,259,479	98	100
	Diginorm	119,529	88		302,443	100		99	100		4,264,234	98	
	Raw	136,176			302,442			99			4,342,602		

In Table 6, we show the total length of the assemblies for *Q*_0_=20 absolute and relative to the length of the reference. In most cases, all assemblies are clearly longer than the reference, with Diginorm by trend causing slightly larger and Bignorm causing slightly shorter assemblies compared to the unfiltered dataset (see Additional file 1: Figure S6 for a box plot).

**Table 6:** Reference length and total length of assemblies for Bignorm with *Q*_0_=20, Diginorm, and the raw datasets

Dataset	Reference	Raw		Diginorm		Bignorm	
	Ref length	Total length	% of ref	Total length	% of ref	Total length	% of ref
Aceto	426,710	750,316	175.80	769,090	180.20	731,850	171.50
Alphaproteo	463,456	405,020	87.40	377,293	81.40	394,979	85.20
Arco	231,937	408,571	176.20	419,403	180.80	380,191	163.90
Arma	1,364,272	2,123,588	155.70	2,131,958	156.30	2,077,037	152.20
ASZN2	3,669,182	4,938,079	134.60	4,930,677	134.40	4,836,216	131.80
Bacteroides	560,676	826,566	147.40	818,799	146.00	792,384	141.30
Caldi	1,961,164	2,044,270	104.20	2,041,841	104.10	2,037,901	103.90
Caulo	423,390	601,709	142.10	616,942	145.70	590,319	139.40
Chloroflexi	863,677	1,317,768	152.60	1,326,848	153.60	1,186,531	137.40
Crenarch	716,004	1,009,122	140.90	1,016,485	142.00	946,606	132.20
Cyanobact	343,353	635,368	185.00	636,876	185.50	591,367	172.20
E. coli	4,639,675	4,896,992	105.50	4,898,422	105.60	4,948,739	106.70
SAR324	4,255,983	4,676,938	109.90	4,674,540	109.80	4,669,774	109.70

Bignorm’s mean phred score is always slightly larger than that of the raw dataset, whereas Diginorm’s is always smaller. For some cases, the difference is substantial; the quartiles for the ratio of Diginorm’s mean phred score to that of the raw dataset are given in Table 7 in the first row.

**Table 7:** Quartiles for comparison of mean phred score, filter and assembler Wall time in %

	Min	$\mathcal {Q}1$	Median	Mean	$\mathcal {Q}3$	Max
Diginorm mean phred score	62	66	74	74	79	89
raw mean phred score						
Bignorm filter time	24	28	31	33	38	46
Diginorm filter time						
Bignorm SPAdes time	4	08	18	26	35	88
Diginorm SPAdes time						

Clearly, our biggest gain is in running time, for the filtering as well for the assembly. Quartiles of the corresponding improvements are given in rows two and three of Table 7.

### IDBA_UD and Velvet-SC

For a detailed presentation of the results gained with IDBA_UD and Velvet-SC, please see “Comparison of different assemblers” section in the Additional file 1. We briefly summarize the results:

- IDBA_UD does not considerably benefit from read filtering, while Velvet-SC clearly does.
- Velvet-SC is clearly inferior to both SPAdes and IDBA_UD, though in some regards the combination of read filtering and Velvet-SC is as good as IDBA_UD.
- SPAdes nearly always produced better results than IDBA_UD, but in median (on unfiltered datasets) IDBA_UD is about 7 times faster than SPAdes.
- SPAdes running on a dataset filtered using Diginorm is approximately as fast as IDBA_UD on the unfiltered dataset while SPAdes on a dataset filtered using Bignorm is roughly 4 times faster.

## Discussion

The quality parameter *Q*_0_ that Bignorm introduces as an innovation to Diginorm has shown to have a strong impact on the number of reads kept, coverage, and quality of the assembly. A reasonable upper bound of *Q*_0_≤25 was obtained by considering the 10th percentile of the coverage (Fig. 2 b). With this constraint in mind, in order to keep a small number of reads, Fig. 1 a suggests 18≤*Q*_0_≤25. Given that N50 for E.coli starts to decline at *Q*_0_=20 (Fig. 3), we decided for *Q*_0_=20 as the recommended value. As presented in detail in Table 4, *Q*_0_=20 gives good assemblies for all 13 cases. The gain in speed is considerable: in terms of the median, we only require 31*%* and 18*%* of Diginorm’s time for filtering and assembly, respectively. This speedup generally comes at the price of a smaller genomic fraction and shorter largest contig, although those differences are relatively slight.

We believe that the increase of the N50 and largest contig for high values of *Q*_0_, which we observe for some datasets just before the breakdown of the assembly (compare for example the results for the Alphaproteo dataset in Fig. 3), is due to the reduced number of branches in the assembly graph: SPAdes, as every assembler, ends a contig when it reaches an unresolvable branch in its assembly graph. As the number of reads in the input decreases more and more with increasing *Q*_0_, the number of these branches also decreases and the resulting contigs get longer.

## Conclusions

For 13 bacteria single cell datasets, we have shown that good and fast assemblies are possible based on only 5*%* of the reads in most of the cases (and on less than 10*%* of the reads in all of the cases). The filtering process, using our new algorithm Bignorm, also works fast and much faster than Diginorm. Like Diginorm, we use a count-min sketch for counting *k*-mers, so the memory requirements are relatively small and known in advance. Our algorithm Bignorm yields filtered datasets and subsequent assemblies of competative quality in much shorter time. In particular, the combination of Bignorm and SPAdes gives superior results to IDBA_UD while being faster. Furthermore, the mean phred score of our filtered dataset is much higher than that of Diginorm.

## Additional file

Additional file 1 See file ’supplement.pdf’ for formal definitions and details on results from different assemblers. (PDF 259 kb)
