# Supplementary material for: An improved filtering algorithm for big read datasets and its application to single-cell assembly
Source: BMC Bioinformatics. 2017 Jul 3;18:324. doi: 10.1186/s12859-017-1724-7 (PMC5496428; doi:10.1186/s12859-017-1724-7)
Supplement: Additional file 1 — See file ’supplement.pdf’ for formal definitions and details on results from different assemblers. (PDF 259 kb) [file 12859_2017_1724_MOESM1_ESM.pdf]

An Improved Filtering Algorithm for Big Read Datasets and its Application to Single-Cell Assembly

— Supplement —

Axel Wedemeyer* Lasse Kliemann† Anand Srivastav
Christian Schielke Thorsten B. Reusch Philip Rosenstiel

June 6, 2017

S1 Formal descriptions

In this section we give a technical description of Bignorm which might be important for further algorithmic enhancements. The level of details are chosen in a way that the essential mathematical parts should be clear.

S1.1 Formal Problem Formulation

Denote by $\mathbb{N} := \{0, 1, 2, \ldots\}$ the set of non-negative integers, and for each $n \in \mathbb{N}$ denote by $[n] := \{1, \ldots, n\}$ the integers from 1 to n (including 1 and n). Denote by $\Sigma := \{\mathsf{A}, \mathsf{C}, \mathsf{G}, \mathsf{T}, \mathsf{N}\}$ the alphabet of nucleotides plus the symbol N used to indicate an undetermined base. By Σ^* we denote all the finite strings over Σ, and for some $k \in \mathbb{N}$ by Σ^k all the strings over Σ of exactly length k. For $v \in \Sigma^*$, denote by $|v| \in \mathbb{N}$ its length and $\bar{v} \in \Sigma^*$ its reverse complement. For $v, w \in \Sigma^*$, we write $v \cong w$ if $|v| = |w|$ and the two strings are equal up to places where either of them has the N symbol.

*axw@informatik.uni-kiel.de
†lki@informatik.uni-kiel.de

The input to the filter algorithm is a *dataset* $D = (n, m, R, Q)$ where for each $i \in [n]$ we have:

- $m(i) \in \mathbb{N}$: a flag for an unpaired ($m(i) = 1$) or paired ($m(i) = 2$) dataset;
- $R(i, s) \in \Sigma^*$ for each $s \in [m(i)]$: the set of *reads* in the dataset;
- $Q(i, s) \in \mathbb{Z}^{|R(i,s)|}$ for each $s \in [m(i)]$: the set of corresponding *phred scores*.

Each read $i \in [n]$ consists of $m(i)$ *read strings* $R(i, 1), \ldots, R(i, m(i))$. For $t \in [|R(i, s)|]$, we denote the nucleotide at position t in read string $R(i, s)$ by $R_t(i, s)$ and its phred score by $Q_t(i, s)$. Note that in terms of read strings, D may contain the "same" read multiple times (perhaps with different quality values), that is, there can be $i \neq j$ such that $R(i) = R(j)$. Hence it is beneficial that we refer to reads by their indices $1, \ldots, n$.

Denote by $x \in \Sigma^*$ the genome from which the reads were obtained and $g := |x|$ its length. (For the purpose of this exposition, we simplify by assuming the genome is a single string.) For each locus $\ell \in [g]$, the *coverage* $c_\ell(D)$ of ℓ with respect to D is informally described as the number of read strings that were or could have been produced by the sequencing machine while reading a part of x that contains locus ℓ. More precisely, for each $v \in \Sigma^*$ define

- $c_\ell(v) := 1$ if there is a substring w of x which contains locus ℓ and satisfies $v \cong w$ or $v \cong \bar{w}$;
- $c_\ell(v) := 0$ otherwise.

Then, we define:

$$c_\ell(D) := \sum_{i=1}^{n} \sum_{s=1}^{m(i)} c_\ell(R(i, s))$$

As result of our algorithm a sub-dataset $D' = (n', m', R', Q')$ of D should be determined such that n' is much smaller than n without losing essential information. The goal is to produce an assembly of similar quality based on D' instead of D. We only consider the natural approach to create D' by making a choice for each $i \in [n]$ whether to include read i in D' or not, so in particular $(R'(1), \ldots, R'(n'))$ will be a sub-vector of $(R(1), \ldots, R(n))$. When we include a read in D', we also say that it is *accepted*, whereas when we exclude it, we call it *rejected*. On an abstract level, a filtered dataset based on D can be specified by giving a set of indices $A \subseteq [n]$ that consists of the accepted reads.

Many popular assemblers, such as SPAdes [1], Platanus [2], or Allpaths-LG [3], work with the *de Bruijn graph* that is based on k-mers. Fix a

parameter $k \in \mathbb{N}$; typically $k \geq 21$ The set of k-mers of a string $v \in \Sigma^*$, denoted $M(v,k) \subseteq \Sigma^k$, is the set of all strings of length k that are substrings of v. Partly we need to consider a k-mer multiple times if it occurs in multiple places in the string, and the corresponding set is denoted:

$$M^*(v,k) := \big\{(\mu,p) \in \Sigma^k \times \mathbb{N}\ ;\ \mu \text{ is a substring of } v \text{ starting at position } p\big\}.$$

For a read $i \in [n]$ and read string $s \in [m(i)]$, define $M(i,s,k) := M(R(i,s),k)$ and $M(i,k) := \bigcup_{s=1}^{m(i)} M(i,s,k)$, so $M(i,k)$ are all the k-mers that occur in any of the $m(i)$ read strings of $R(i)$. Denote also $M^*(i,s,k) := M^*(R(i,s),k)$.

S1.2 Formal description of CMS

Bignorm, like Diginorm, is based on the count-min sketch (CMS) for counting k-mers. CMS is a probabilistic data structure for counting objects from a large universe. We give a brief and abstract description. Let $a = (a_1,\dots,a_N) \in \mathbb{N}^N$ be a vector, given implicitly as a sequence of updates of the form (p,Δ) with $p \in [N]$ and $\Delta \in \mathbb{N}$. Each update (p,Δ) modifies a in the way $a_p := a_p + \Delta$; where initially $a = (0,\dots,0)$. If $\Delta = 1$ in each update, then an interpretation of the vector a is that we count how many times we observe each of the objects identified by the numbers in $[N]$. If N is large, e.g. if N is the number 4^k of all possible k-mers (we do not count k-mers with N symbols), then we may not be able to store a in RAM. (For example, the typical choice of $k = 21$ brings a into terabyte range; in our experiments we use $k = 32$.) Instead we fix two parameters: the *width* $m \in \mathbb{N}$ and the *depth* $t \in \mathbb{N}$ and store a matrix of $m \cdot t$ *CMS counters* $c_{p,q}$ with $p \in [m]$ and $q \in [t]$. Moreover, we randomly draw t hash functions $h_1,\dots,h_t$ from a universal family. Each h_q maps from $[N]$ to $[m]$. Initially, all counters in the matrix are zero. Upon arrival of an update (p,Δ), for each row $q \in [t]$ we update $c_{h_q(p),q} := c_{h_q(p),q} + \Delta$. That is, for each row q, we use the hash function h_q to map from the larger space $[N]$ (from which the index p comes) to the smaller space $[m]$ of possible positions in the row. Denote

$$\widehat{a}_p := \min\{c_{h_1(p),1},\dots,c_{h_t(p),t}\}\ . \tag{1}$$

Then, it can be proved [4] that $\widehat{a}_p$ is an estimate of a_p in the following sense: clearly $a_p \leq \widehat{a}_p$, and with probability at least $1 - e^{1-t}$ we have $\widehat{a}_p \leq \frac{e}{m-1}\sum_{j=1}^{N} a_j$. The probability is over the choice of hash functions. For example, choosing $t := 10$ is enough to push the error probability, upper-bounded by e^{1-t}, below 0.013%.

In our application, $N = 4^k$ is the number of possible k-mers (without N symbols) and we implement a bijection $\beta : \Sigma^k \longrightarrow [N]$, so we can

identify each k-mer μ by a number $\beta(\mu) \in [N]$. Upon accepting some read i, we update the CMS counters using all the updates of the form $(\beta(\mu), 1)$ with $\mu \in M(i,k)$ not containing the N symbol, that is, for each such μ we increase the count $\beta(\mu)$ by $\Delta = 1$. Then when all the reads $1, \ldots, i-1$ have been processed, the required count $c(\mu, i)$ corresponds to the entry $a_{\beta(\mu)}$ in the vector a used in the description of CMS, and for the estimate $\widehat{c}(\mu, i)$, we can use the estimate $\widehat{a}_{\beta(\mu)}$ as given in (1).

S1.3 Formal description of algorithm

We give a detailed description of our enhancements (i) to (iv) that were briefly lined out on page ??. Although most of the settings are generic, in some places we assume that data comes from the Illumina.

We start with (i), (ii), and (iii). Fix a read $i \in [n]$ and a read string $s \in [m(i)]$. Recall that for each $t \in [\,|R(i,s)|\,]$ the nucleotide $R_t(i,s)$ at position t in the read string $R(i,s)$ is associated with a quality value $Q_t(i,s)$ known as *phred score*. We want to assign a single value $Q(i,s,\mu,p)$ to each $(\mu,p) \in M^*(i,s,k)$. We do so by taking the minimum phred score over the nucleotides in μ when aligned at position p, that is:

$$Q(i,s,\mu,p) := \min_{p \le t \le p+k-1} Q_t(i,s)$$

(μ occurs on the right-hand side only implicitely through its length k).

Fix the following parameters:

- N-*count threshold* $N_0 \in \mathbb{N}$, which is 10 by default;
- *quality threshold* $Q_0 \in \mathbb{Z}$, which is 20 by default;
- *rarity threshold* $c_0 \in \mathbb{N}$, which is 3 by default;
- *abundance threshold* $c_1 \in \mathbb{N}$, which is 20 by default;
- *contribution threshold* $B \in \mathbb{N}$, which is 3 by default.

When our algorithm has to decide whether to accept or reject a read $i \in [n]$, it performs the following steps. If the number of N symbols counted over all $m(i)$ read strings in i is larger than N_0, the read is rejected. Otherwise, for each $s \in [m(i)]$ define the set of *high-quality k-mers*:

$$H(s) := \big\{(\mu,p) \in M^*(i,s,k) : (Q_0 \le Q(i,s,\mu,p)) \\ \text{and } (\mu \text{ does not contain N})\big\}.$$

We determine the *contribution* of $R(i,s)$ to k-mers of different frequencies:

$$b_0(s) := |\{(\mu,p) \in H(s)\ ;\ \widehat{c}(\mu,i) < c_0\}|,$$
$$b_1(s) := |\{(\mu,p) \in H(s)\ ;\ c_0 \le \widehat{c}(\mu,i) < c_1\}|$$

Note that the frequencies are determined via CMS counters and do not consider the position p at which the k-mer is found in the read string. The read i is accepted if and only if at least one of the following conditions is met:

$$b_0(s) > k \text{ for at least one read string } s, \tag{2}$$

$$\sum_{s=1}^{m(i)} b_1(s) \geq B. \tag{3}$$

If the read is accepted, then for each $\mu \in M(i,k)$ the corresponding CMS counter is incremented, provided that μ does not contain the N symbol. Afterwards processing of the next read starts.

The motivation for condition (2) is as follows. According to [5], most errors of the Illumina platform are single substitution errors and the probability of appearance of an erroneous k-mer in the genome, caused by an incorrect reading of a nucleotide, is quite low. Thus, k-mers produced by single substitution errors are likely to have very small counter values in the CMS (less than c_0 times) and can be considered as rare k-mers. One such error can only effect at most k k-mers. So if we count more than k rare k-mers, they must be the result of factors other than one single substitution error. If we assume that the probability of multiple single substitution errors in a read is smaller than the probability of error-free rare k-mers, we should accept this read.

Condition (3) says that in the read i, there are enough (namely at least B) k-mers where each of them is too frequent to be a read error (CMS counters at least c_0) but not that abundant that it should be considered redundant (CMS counters less than c_1).

This concludes the description of (i), (ii), and (iii), particularly how we analyze the counts in $C(i,s) = (\widehat{c}(\mu,i))_{\mu \in M(i,s,k)}$ for each read i and $s \in [m(i)]$, how we incorporate quality information, and how we handle the N symbol.

Finally, to accomplish (iv), we wrote a multi-threaded implementation completely in the C programming language. The parallel code uses OpenMP. For comparison, the implementation of the original Diginorm algorithm (included in the khmer-package [6]) features a single-threaded design and is written in Python and C++; strings have to be converted between Python and C++ at least twice.

S2 Comparison of different assemblers

To investigate how digital normalization affects other assemblers, we ran IDBA_UD and Velvet-SC on

- the unfiltered dataset

Assembler	Normalization	
	Diginorm	Bignorm
SPAdes	8.2×	28.0×
IDBA_UD	3.8×	18.0×
Velvet-SC	65.2×	255.2×

Table S1: Median speed-up by assembler and normalization algorithm

- the dataset filtered with Diginorm and
- the dataset filtered with Bignorm, Q_0 set to 20

for each of the 13 test datasets.

For comprehensibility, we used the measurements for SPAdes on the unfiltered dataset as a reference. We present box plot diagrams for the measures largest contig, N50, genome fraction, total length, and assembler runtime (Walltime), grouped by assembler and by normalization used.

For largest contig and N50, IDBA_UD does not benefit from normalization while Velvet-SC clearly does. Both assemblers however, appear to be inferior to SPAdes (regardless of normalization). The results of Velvet-SC on normalized data are comparable to IDBA_UD (see Supplement Figure 1 and 2).

For genome fraction, both IDBA_UD and Velvet-SC profit from normalization, but Velvet-SC always performs worse than IDBA_UD, whose results are bested by SPAdes (see Supplement Figure 3).

For total length of the assembly, IDBA_UD (like SPAdes) does profit from normalization with Diginorm, but using Bignorm led to a deterioration. Velvet-SC benefits from both normalization algorithms, but its results are still inferior to IDBA_UD and SPAdes (see Supplement Figure 4).

For the assembler runtime (Walltime), all assemblers get substantially faster when using normalization (see Supplement Figure 5. The diagram shows that SPAdes on a dataset normalized with Bignorm is always faster that IDBA_UD on the same, unfiltered dataset. Remarks:

- the y-axis is logarithmised for an informative plot
- the runtime given for Velvet-SC is the sum of the runtimes of `velveth` and `velvetg`

See Table S1 for the median speed-up by assembler and normalization algorithm.

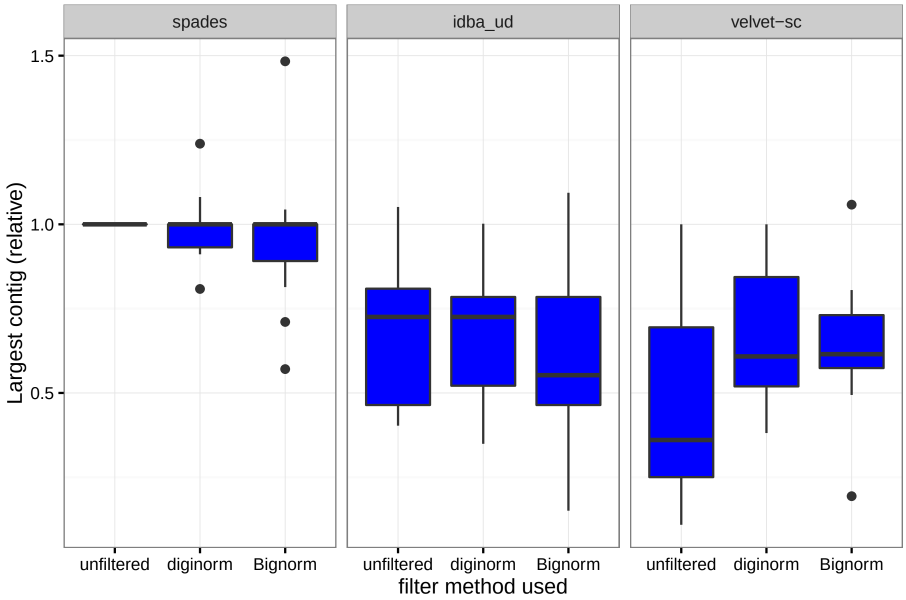

Supplement Figure 1: Comparison of largest contig for SPAdes, IDBA_UD and Velvet-SC relative to SPAdes on unfiltered dataset.

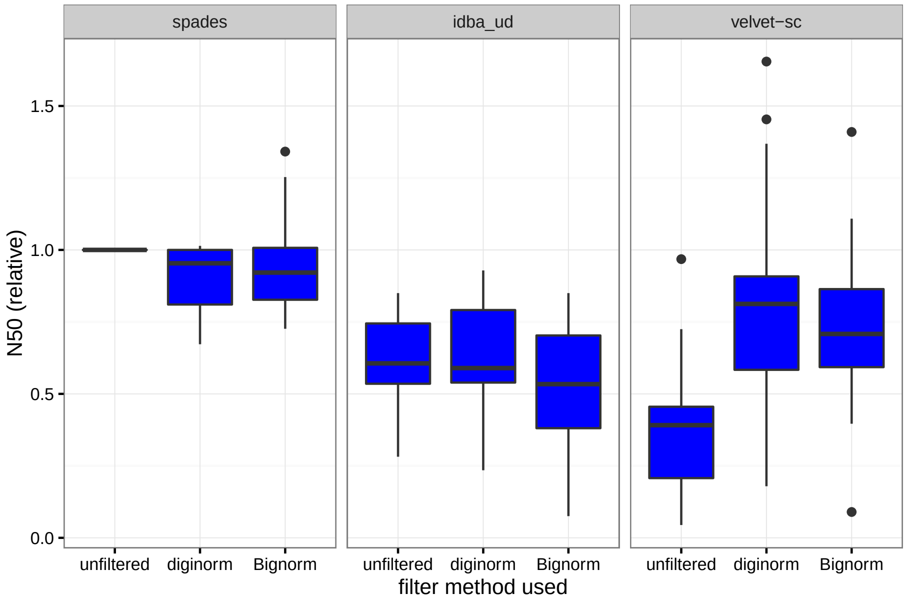

Supplement Figure 2: Comparison of N50 for SPAdes, idba_ud and velvet-sc relative to SPAdes on unfiltered dataset.

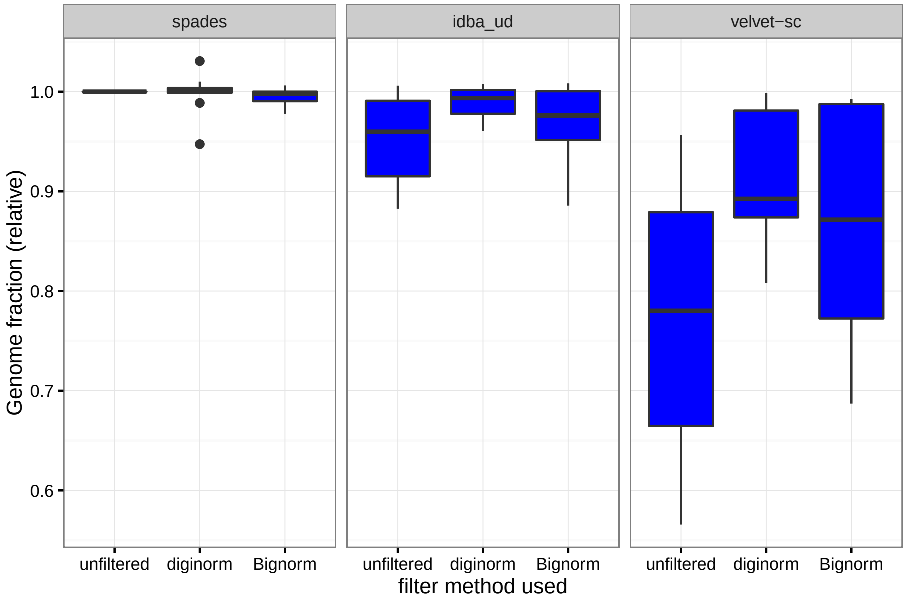

Supplement Figure 3: Comparison of genome fraction for SPAdes, idba_ud and velvet-sc relative to SPAdes on unfiltered dataset.

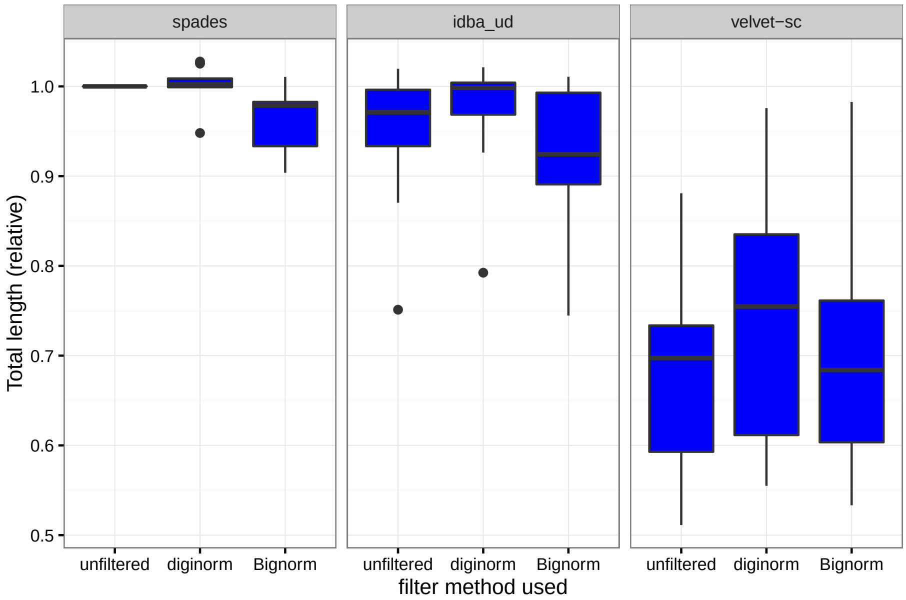

Supplement Figure 4: Comparison of total length of assemblies for SPAdes, idba_ud and velvet-sc relative to SPAdes on unfiltered dataset.

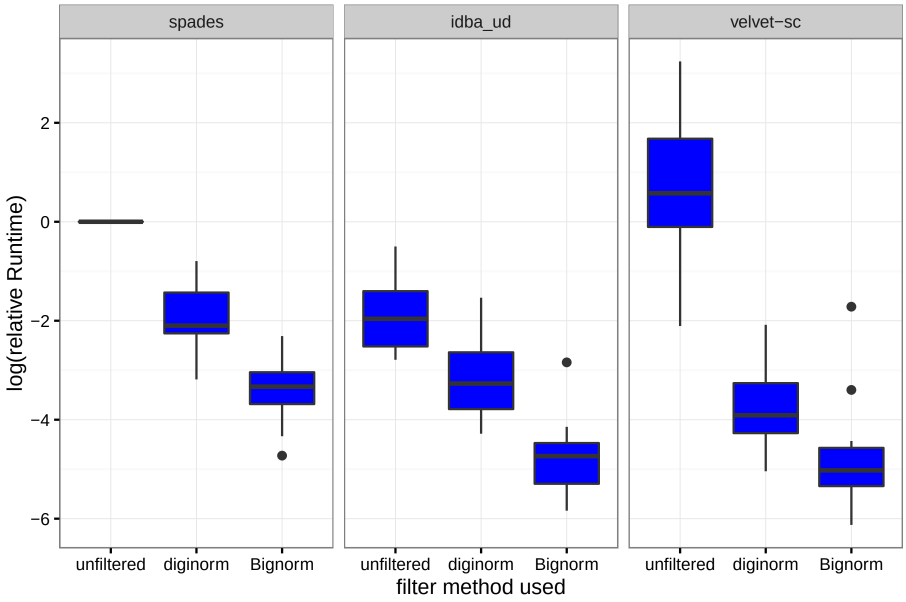

Supplement Figure 5: Comparison of log of runtime for SPAdes, idba_ud and velvet-sc relative to SPAdes on unfiltered dataset.

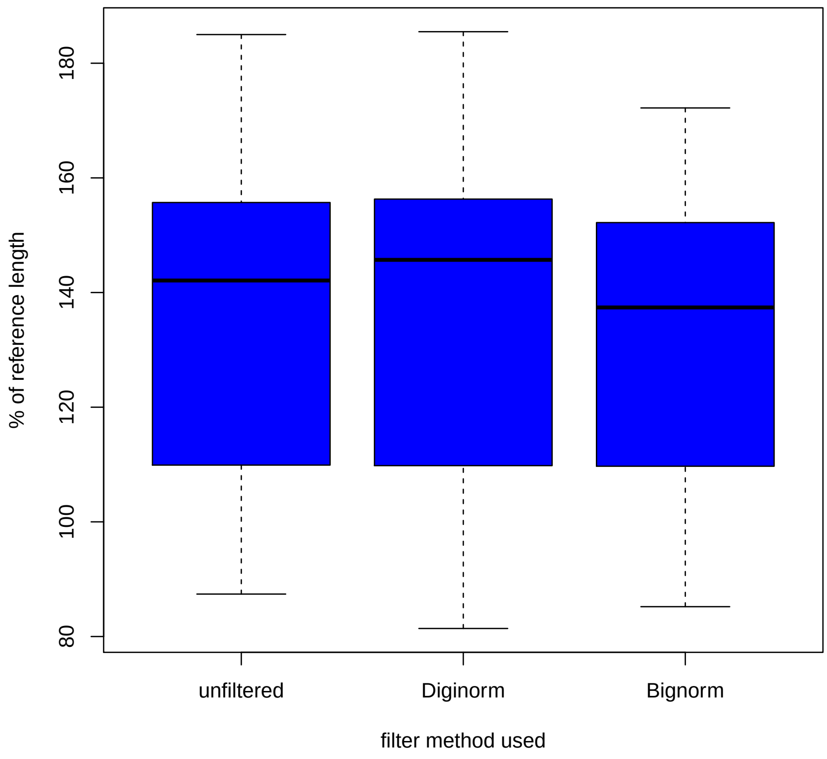

Supplement Figure 6: Comparison of the total length of the SPAdes assemblies by filter method used relative to the length of the reference

S3 Total length of Assemblies

The distribution of the total length of the assemblies done with SPAdes and Q_0 set to 20 are shown in Supplement Figure 6. For the plot, all measures were divided by the length of the related reference genome as provided by JGI / UCD.

S4 Quality plots of datasets

As Bignorm uses the phred score in its decision function, it is not surprising that low quality datasets give low quality normalizations. Especially the decline rate of the phred score over the read position, as shown in Supplement Figure 7, turned out to have a big impact on the result of the normalization.

References

[1] Bankevich, A., Nurk, S., Antipov, D., Gurevich, A.A., Dvorkin, M., Kulikov, A.S., Lesin, V.M., Nikolenko, S.I., Pham, S., Prjibelski, A.D., Pyshkin, A.V., Sirotkin, A.V., Vyahhi, N., Tesler, G., Alekseyev, M.A., Pevzner, P.A.: SPAdes: A New Genome Assembly Algorithm and Its Applications to Single-Cell Sequencing. Journal of Computational Biology **19**(5), 455–477 (2012). doi:10.1089/cmb.2012.0021

[2] Kajitani, R., Toshimoto, K., Noguchi, H., Toyoda, A., Ogura, Y., Okuno, M., Yabana, M., Harada, M., Nagayasu, E., Maruyama, H., Kohara, Y., Fujiyama, A., Hayashi, T., Itoh, T.: Efficient de novo assembly of highly heterozygous genomes from whole-genome shotgun short reads. Genome Research, 1384–1395 (2014). doi:10.1101/gr.170720.113

[3] Gnerre, S., Maccallum, I., Przybylski, D., Ribeiro, F.J., Burton, J.N., Walker, B.J., Sharpe, T., Hall, G., Shea, T.P., Sykes, S., Berlin, A.M., Aird, D., Costello, M., Daza, R., Williams, L., Nicol, R., Gnirke, A., Nusbaum, C., Lander, E.S., Jaffe, D.B.: High-quality draft assemblies of mammalian genomes from massively parallel sequence data. Proc Natl Acad Sci U S A **108**(4), 1513–1518 (2011). doi:10.1073/pnas.1017351108

[4] Cormode, G., Muthukrishnan, S.: An improved data stream summary: the count-min sketch and its applications. Journal of Algorithms **55**(1), 58–75 (2005). doi:10.1016/j.jalgor.2003.12.001

[5] Kelley, D.R., Schatz, M.C., Salzberg, S.L.: Quake: quality-aware detection and correction of sequencing errors. Genome Biol **11**(11), 1–13 (2010). doi:10.1186/gb-2010-11-11-r116

[6] Crusoe, M., Edvenson, G., Fish, J., Howe, A., McDonald, E., Nahum, J., Nanlohy, K., Ortiz-Zuazaga, H., Pell, J., Simpson, J., Scott, C., Srinivasan, R.R., Zhang, Q., Brown, C.T.: The khmer software package: enabling efficient sequence analysis, 1–3 (2014). doi:10.6084/m9.figshare.979190

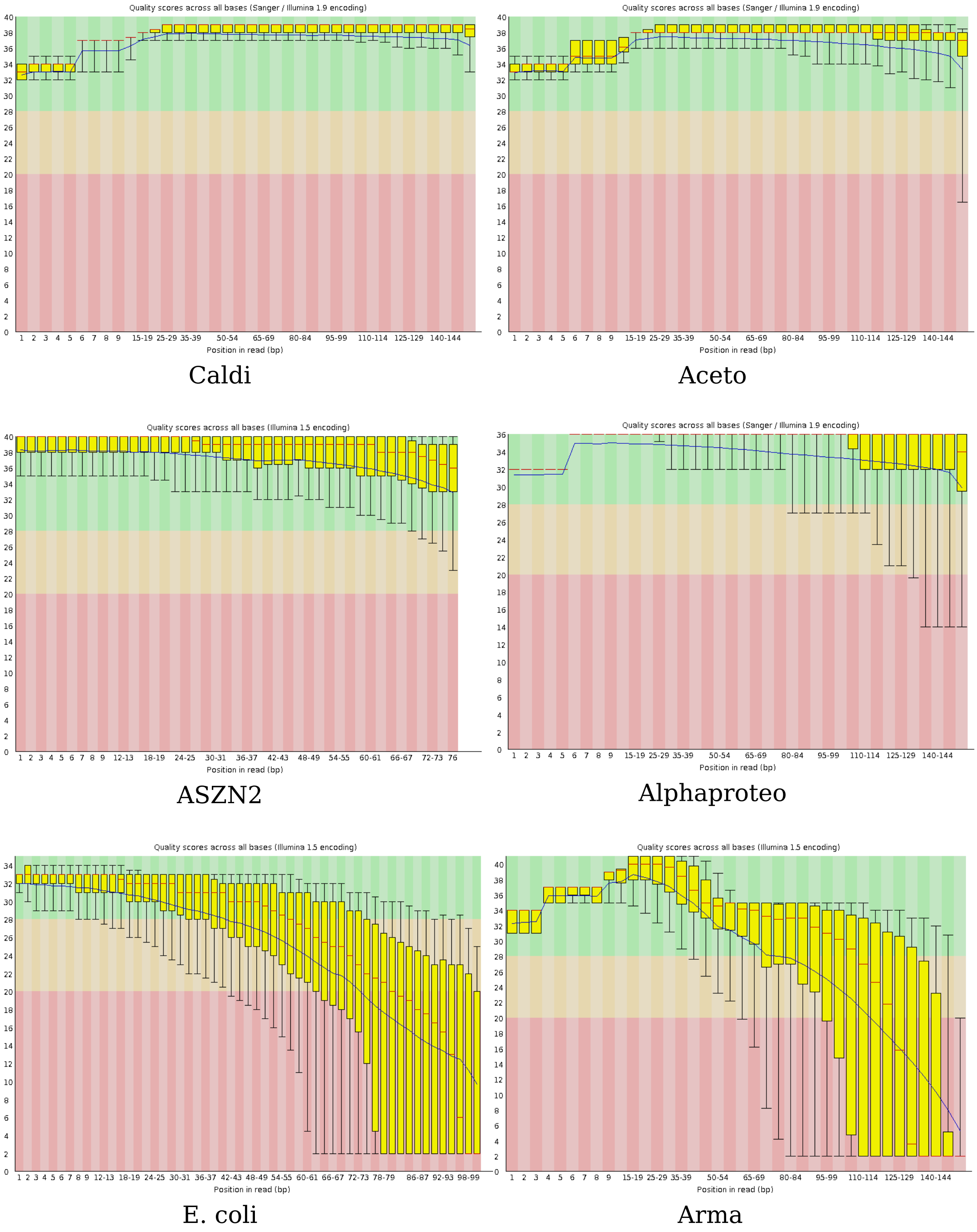

Supplement Figure 7: Quality plots of selected datasets, generated using the `FastQC` tool and ordered by decreasing quality
